# Does Restricting Pack Size of Paracetamol (Acetaminophen) Reduce Suicides?

**DOI:** 10.1371/journal.pmed.0040152

**Published:** 2007-04-03

**Authors:** Nicholas A Buckley, David Gunnell

## Abstract

The authors discuss a new study that examined the change in deaths attributed to paracetamol poisoning in England and Wales in the six years before and after a legislated reduction in the maximum pack size.

Public health interventions are usually implemented without any attempt to prospectively evaluate them with an experimental research design. Thus the only way to evaluate the outcome of the intervention is to describe what happened before and after its implementation. In a new study published in *PLoS Medicine*, Morgan et al. have examined the change in deaths attributed to paracetamol (acetaminophen) poisoning in England and Wales in the six years before and after a legislated reduction in the maximum pack size [1]. The average number of deaths preceding the regulation was 212/year and afterwards it fell to an average of 154/year (see Figure 1 and Table 1 in [1]). Therefore, readers could be forgiven for being puzzled that the authors have declared that they found little evidence to suggest that the regulations caused the reduction.

The authors did show statistically significant changes in deaths attributed to paracetamol poisoning after the legislation compared to the preceding six years before the legislation (Table 2 in [1]). But they also assessed whether the observed changes over time were unique to paracetamol. They compared the changes against the number of poisoning deaths involving compound paracetamol (not covered by the regulations), aspirin, antidepressants, and against the number of non-poisoning suicide deaths, over the same time period. It is the lack of statistically significant and consistent changes in the relative rates of deaths for paracetamol versus coincidental changes for other drugs (Table 4 in [1]) that lead the authors to conclude that the regulations may have had no effect.

## Was the Conclusion Justified?

The changes seen with each of the comparator drugs in this study might each have an explanation that would cast the regulatory effect in a more favourable light.

For a start, the regulations also applied to aspirin—so it is hardly surprising that a similar reduction was noted for aspirin fatalities at the same time. Indeed the reductions in death from both paracetamol and from aspirin could be considered as further evidence of the effectiveness of the regulations.

Second, it is likely that some deaths attributed to paracetamol compounds are deaths where there has also been substantial co-ingestion of non-compound paracetamol, since people commonly ingest more than one medication in overdose. Deaths in these circumstances due to paracetamol hepatotoxicity may still be classified as due to paracetamol compound, so the reduction in paracetamol compound deaths may, in part, have resulted from the legislation [2]. Conversely, deaths due to the other components of compound preparations may be misclassified as deaths due to paracetamol. While coronial records may have improved, 25 years ago over a quarter of deaths classified as due to paracetamol were on review found to be due to paracetamol/propoxyphene combinations [2]. The rate of these deaths obviously cannot be modified by regulating paracetamol.

Third, the changes in deaths from antidepressant overdose seem likely to reflect changes in antidepressant prescribing over the last decade [3]. An important trend in such prescribing is the increasing use of selective serotonin reuptake inhibitors and other new agents that are less toxic in overdose than older antidepressants [4], particularly for new patients and others at higher risk of suicide.

Finally, changes in overall suicide rates may reflect reductions in carbon monoxide poisoning due to the introduction of catalytic converters in cars [5]. So while Morgan and colleagues' new study raises the possibility that the changes in paracetamol poisoning deaths over time may be unrelated to the legislation, and may in fact be due to other more general changes in suicidal behaviour, the argument is far from conclusive.

All the changes in suicide rate shown in Figure 1 in [1] could be reinterpreted to reflect the effects of reductions in the lethality of particular modes of suicide, rather than being an indication of the ineffectiveness of legislation on pack size. Historically, the most dramatic reductions in suicide rates have reflected changes in lethality of method rather than a reduction in attempts. One oft-quoted example is the 80%–87% fall in gas-poisoning deaths between 1963 and 1971 in England and Wales with the replacement of coal gas with natural gas [6]. Even more impressive is the halving of total suicide rates in Sri Lanka over ten years coinciding with the banning of a small number of highly lethal pesticides (Gunnell et al., unpublished data).

Frequentist statistics, as used by Morgan et al., test whether a null hypothesis can be rejected from the data in the study. The researchers have not proven there was no effect from the legislation; they simply were unable to confidently reject the possibility that this difference over the next six years was simply due to chance.

## Other Studies on Drug Packaging and Self Harm

Morgan and colleagues' study should not be examined in isolation. While the effect of safety packaging in reducing lethal childhood poisoning is established, there have been only a few other instances where the effect of such regulatory changes on adult poisoning have been evaluated [7,8]. However, many researchers have taken the opportunity to look at the effect of restrictions on paracetamol pack size [9–19]. These studies have generally shown small but favourable effects—in some cases reaching statistical significance and sometimes not. Two studies from Scotland showed no change whatsoever [9,10].

It seems most likely that the pack size regulations did change the pattern of paracetamol poisoning but that changes were far more modest than hoped. Ingestion of eight grams of paracetamol (the maximum amount allowed in a pack from non-pharmacy retail outlets) should rarely lead to toxicity and ingestion of 16 grams of paracetamol (the maximum pack size available from pharmacies) should rarely lead to death. So the reasons for the very modest reduction in deaths found in these studies may be due to failures in implementation rather than any inherent flaw in the concept. A study in London three years after the regulations were introduced found that 46% of people presenting with overdose had purchased potentially toxic amounts of paracetamol in a manner contravening the spirit of the 1998 legislation and the Medicines and Healthcare Products Regulatory Agency's recommended voluntary restraints on multiple packet sales [20].

## Implications for Drug Regulators

What is the lesson for regulators in the UK and elsewhere from these studies on paracetamol regulation in the UK? We think they provide some further evidence that changes in drug availability and packaging can lead to reductions in deaths from self harm. However, the changes may be much less than anticipated if they cannot be enforced.

Morgan et al.'s study also indicates the benefits of having national databases on deaths from poisoning. These databases could be further strengthened by having more information routinely collected and regularly audited for accuracy. At the moment such databases are more than adequate for determining priorities for public health interventions but less than ideal for evaluating these same interventions. We would heartily endorse the authors' proposal to ensure that prospective evaluation is an integral part of such public health interventions [12]. When this happens, we will know that evidence-based public health is more than just a slogan.
